# Prevalence and clinical features of autosomal dominant and recessive *TMC1*-associated hearing loss

**DOI:** 10.1007/s00439-021-02364-2

**Published:** 2021-09-14

**Authors:** Shin-ya Nishio, Shin-ichi Usami

**Affiliations:** Department of Hearing Implant Sciences, Shinshu University School of Medicine, 3-1-1 Asahi, Matsumoto, 390-8621 Japan

## Abstract

*TMC1* is a causative gene for both autosomal dominant non-syndromic hearing loss (DFNA36) and autosomal recessive non-syndromic hearing loss (DFNB7/11). To date, 125 pathogenic variants in *TMC1* have been reported. Most of the *TMC1* variants are responsible for autosomal recessive hearing loss, with only 8 variants reported as causative for DFNA36. Here, we reported the prevalence of *TMC1*-associated hearing loss in a large non-syndromic hearing loss cohort of about 12,000 subjects. As a result, we identified 26 probands with *TMC1*-associated hearing loss, with the estimated prevalence of *TMC1*-associated hearing loss in the Japanese hearing loss cohort being 0.17% among all patients. Among the 26 probands with *TMC1*-associated hearing loss, 15 cases were identified from autosomal dominant hearing loss families. Based on the audiometric data from the probands, family members and previously reported cases, we evaluated hearing deterioration for DFNA36 patients. In addition, we performed haplotype analysis for 11 unrelated autosomal dominant hearing loss families carrying the same variant *TMC1*: NM_138691:c.1627G > A:p.Asp543Asn. The results clearly indicated that the same haplotype was present despite the families being unrelated, supporting the contention that this variant occurred by founder mutation.

**Supplementary Information:**

The online version contains supplementary material available at 10.1007/s00439-021-02364-2.

## Introduction

Hearing loss is one of the most common sensory disorders and, currently, approximately 120 genes have been reported as causative for non-syndromic hearing loss (The Hereditary Hearing Loss Homepage). *TMC1* is a causative gene for both autosomal dominant non-syndromic hearing loss (ADNSHL) and autosomal recessive non-syndromic hearing loss (ARNSHL) as first reported by Kurima et al (2002). The encoding protein transmembrane channel-like protein 1 is highly expressed in the tips of stereocilia and plays a crucial role in mechano-electro-transduction (Liu et al. 2020).

*TMC1* variants are a relatively common genetic cause of non-syndromic hearing loss, and accounts for 3.4% (19/557) of Pakistani ARNSHL patients (Kitajiri et al. 2007a, b), 2.4% (3/125) of Chinese ARNSHL patients (Yang et al. 2013), 0.69% (3/433) of Chinese hearing loss patients (Yuan et al. 2020), 3.1% (4/131) of Western European *GJB2-*negative ARNSHL patients (Sommen et al. 2016), 0.5% (1/200) of Dutch hearing loss patients (Seco et al. 2017), 0.8% (4/491) of Palestinian hearing loss patients (Abu Rayyan et al. 2020), 0.5% (1/197) of Czech hearing loss patients (Safka Brozkova et al. 2020), 4.3% (4/93) to 8.1% (7/86) of Turkish ARNSHL (Kalay et al. 2005; Sirmaci et al. 2009), 5.9% (5/85) of Tunisian ARNSHL (Tlili et al. 2008) and 0.9% (10/1119) of American (Sloan‐Heggen et al. 2016) hearing loss patients. Most cases of *TMC1*-associated hearing loss are identified as autosomal recessive inherited hearing loss, and only limited cases are identified as autosomal dominant. The clinical phenotypes of *TMC1*-associated hearing loss differ according to the inheritance mode. *TMC1*-associated ARNSHL cases show congenital severe-to-profound hearing loss, whereas ADNSHL cases show late-onset progressive hearing loss with predominant deterioration in the higher frequencies. To date, 125 pathogenic variants in *TMC1* have been reported (HGMD Professional). Among the 125 pathogenic variants, only 8 variants were reported as causative for ADNSHL (DFNA36). The *TMC1* gene variants associated with ADNSHL are p.Ile266Thr (Sloan-Heggen et al. 2016), p.Ser320Arg (Hassan et al. 2015), p.Tyr381Asn (Likar et al. 2018), p.Gly417Arg (Yang et al. 2010), p.Met418Lys (Zhao et al. 2014; Wang et al. 2018), p.Asp543Asn (Moteki et al. 2016), p.Asp572Asn (Kurima et al. 2002; Wang et al. 2018; Ramzan et al. 2020), and p.Asp572His (Kitajiri et al. 2007a, b). However, there is some conflict regarding the pathogenicity of the p.Asp572His variants (Azaiez et al. 2018). In addition, the p.Ile266Thr variant and p.Tyr381Asn variant were also reported as causative for *TMC1*-associated ARNSHL (Wang et al. 2018; Sommen et al. 2016). Therefore, only five variants identified from 8 families are reliably known to be the genetic cause of *TMC1*-associated ADNSHL. Based on this limited number of cases, the overall picture regarding the clinical phenotypes of *TMC1*-associated ADNSHL remains unclear.

Recently, autosomal dominant *TMC1*-associated hearing loss has received special attention as a candidate for gene therapy. A mouse model of *TMC1*-related hearing loss (Beethoven mice), generated by ENU mutagenesis, showed autosomal dominant inherited progressive hearing loss (Vreugde et al. 2002). This mouse model carries the *Tmc1*:c.1235T > A:p.Met412Lys variant, and subsequent to this report, ADNSHL patients with an orthologous *TMC1* variant (*TMC1* c.1253T > A:p.Met418Lys) were reported (Zhao et al. 2014). As the Beethoven mice showed a similar phenotype (progressive hearing loss with predominant deterioration in the higher frequencies) to human patients and carried the orthologous mutation identified in human ADNSHL patients, this mouse model is widely used for translational research for gene therapy (Askew et al. 2015; Shibata et al. 2016; Yoshimura et al. 2019; Gao et al. 2018; Nist-Lund et al. 2019; György et al. 2019; Wu et al. 2021). However, prior to the clinical application of gene therapies, the detailed phenotypes and prevalence information are essential.

In this study, we sought to (1) elucidate the prevalence of hearing loss (HL) caused by *TMC1* variants in a large cohort of non-syndromic hearing loss patients, (2) analyze the rate of HL deterioration in *TMC1*-associated ADNSHL patients, and (3) carry out haplotype analysis of the *TMC1*: NM_138691:c.1627G > A:p.Asp543Asn variant identified from 11 unrelated ADNSHL families to confirm whether the mutation occurred by founder mutation or in a mutational hotspot.

## Methods

### Subjects

We performed target re-sequencing analysis for 12,139 Japanese non-syndromic sensorineural hearing loss patients and controls (2462 autosomal dominant or mitochondrial inheritance cases, 6912 autosomal recessive inheritance or sporadic cases, 2220 unknown family history cases, 212 cases with unilateral hearing loss, and 333 normal hearing control subjects) from 90 otorhinolaryngology departments spread across Japan enrolled in this study. In addition, we also analyzed 187 cochlear implant patients or electric acoustic stimulation patients enrolled from 10 cochlear implantation centers listed below: Antwerp University Hospital, Belgium (Prof. Paul Van de Heyning); Hospital Universitario La Paz, Spain (Prof. Javier Gavilán); Klinikum der Universität München, German (Prof. Joachim Müller); Karolinska University Hospital, Sweden (Prof. Eva Karltorp); Institute of Physiology and Pathology of Hearing, Poland (Dr. Henryk Skarzynski and Dr. Piotr Skarzynski); King Abdulaziz University Hospital, Saudi Arabia (Prof. Abdulrahman Hagr), ENT Super Speciality Institute and Research Center, India (Dr. Manikoth Manoj); University of Western Australia, Australia (Prof. Gunesh Rajan); Kansas University, USA (Prof. Hinrich Staecker); and Allende Sanatorio, Argentina (Dr. Mario Zernotti).

Informed written consent was obtained from all subjects (or guardians in the case of minors) prior to participation. This study was approved by the Shinshu University Ethics Committee (Approval number: 576) and the respective ethics committees of all other participating institutions.

### Next-generation sequencing and bioinformatic analysis

Next-generation sequencing was performed for the 63 genes reported to cause non-syndromic hearing loss as described in a previous report (Nishio et al. 2015). In brief, amplicon libraries were prepared using the Ion AmpliSeq Custom Panel, with the Ion AmpliSeq Library Kit 2.0 and the Ion Xpres Barcode Adapter 1-96 Kit (Life Technologies) according to the manufacturer’s instructions. After amplicon library preparation, equal amounts of libraries for 45 patients were pooled for 1 sequence reaction and next-generation sequencing was performed by Ion Proton system with an Ion P1 chip or Ion S5 system with an Ion 540 chip according to the manufacturer’s instructions. The sequence data were aligned to the human reference genome sequence (build GRCh37/hg19) by the Torrent Mapping Alignment Program (TMAP) and, subsequently, DNA variants were piled up with the Torrent Variant Caller plug-in software including in the Torrent Suit (Life Technologies).

The effects of the variants were analyzed using ANNOVAR software (Wang et al. 2010). The missense, nonsense, insertion/deletion, and splicing variants were selected among the identified variants. Variants were further selected as < 1% of several control database including the 1000 genome database (http://www.1000genomes.org/), the 6500 exome variants (http://evs.gs.washington.edu/EVS/), The Genome Aggregation Database (https://gnomad.broadinstitute.org), the human genetic variation database (dataset for 1208 Japanese exome variants) (http://www.genome.med.kyoti-u.ac.jp/SnpDB/index.html), the 8300 Japanese genome variation database (https://jmorp.megabank.tohoku.ac.jp/202102/) and the 333 in-house Japanese normal hearing controls. All filtering procedures were performed using original database software described previously (Nishio and Usami 2017). The pathogenicity of the identified variants was evaluated in accordance with the American College of Medical Genetics (ACMG) standards and guidelines (Richards et al. 2015) with the ClinGen hearing loss clinical domain working group expert specification (Oza et al. 2018). We performed Sanger sequencing analysis to validate the identified variants using PCR and exon-specific custom primers according to the manufacturer’s instructions. All primers were designed using the web version Primer 3 plus software (http://www.bioinformatics.nl/cgi-bin/primer3plus/primer3plus.cgi).

### Haplotype analysis

The haplotype pattern within the 3 Mbp region surrounding the frequent Japanese variation *TMC1*: NM_138691:c.1627G > A identified in this study was analyzed using a set of 47 single-nucleotide polymorphisms (SNPs) (21 sites for upstream and 26 sites for downstream). For this analysis, we selected 15 individuals (including 11 affected and 4 un-affected family members) from 5 families. Haplotype analysis was performed by Sanger sequencing. The mutation-linked haplotype was determined by family member segregation analysis with multiple family member samples, and compared among unrelated families with the same mutations.

## Results

### Identified variants, prevalence, and the clinical features of TMC1-associated hearing loss

As a result of the large cohort next-generation sequencing analysis, we identified 26 probands with *TMC1*-associated hearing loss (Table 1 and Supplemental Fig. 1). The pedigrees and audiometry results are shown in Supplemental Fig. 1. Among the 26 probands, 15 were identified from ADNSHL or maternally inherited cases, whereas 11 were identified from ARNSHL or sporadic cases. No other candidate pathogenic variants in the other 62 deafness genes were identified from these 26 probands. When we restricted analysis to Japanese bilateral non-syndromic hearing loss patients, the prevalence of *TMC1*-associated hearing loss was 0.17% (20/11,594) for all patients, 0.61% (15/2462) for ADNSHL and 0.07% (5/6912) for ARNSHL or sporadic hearing loss cases.

**Table 1:** *TMC1*-associated hearing loss cases identified in this study

ID	Inheritance	Variant 1		Variant 2		Ethnicity	Type of HL	Severity of HL	Progression	Tinnitus	Vertigo
		Base change	AA change	Base change	AA change						
O4886	AD	c.1627G > A	p.Asp543Asn			Japanese	Flat	Profound	Yes	Yes	Yes
O4091	AD	c.1627G > A	p.Asp543Asn			Japanese	Flat	Profound	Yes	Yes	No
O5030	AD	c.1627G > A	p.Asp543Asn			Japanese	Flat	Moderate	Yes	Yes	No
HL2672	AD	c.1627G > A	p.Asp543Asn			Japanese	Flat	Profound	Yes	Yes	No
O0487	AD	c.1627G > A	p.Asp543Asn			Japanese	NA	Profound	Yes	NA	NA
HL6536	AD	c.1627G > A	p.Asp543Asn			Japanese	High freq	Severe	Yes	NA	NA
HL9117	AD	c.1627G > A	p.Asp543Asn			Japanese	High freq	Moderate	Yes	No	No
HL9205	AD	c.1627G > A	p.Asp543Asn			Japanese	NA	Profound	Yes	NA	NA
HL9597	AD	c.1627G > A	p.Asp543Asn			Japanese	High freq	Severe	Yes	Yes	No
HL4994	AD	c.1627G > A	p.Asp543Asn			Japanese	NA	NA	NA	NA	NA
HL6717	AD	c.1627G > A	p.Asp543Asn			Japanese	NA	NA	NA	NA	NA
HL3819	AD	c.1714G > A	p.Asp572Asn			Japanese	High freq	Moderate	NA	NA	NA
HL4498	AD	c.1714G > A	p.Asp572Asn			Japanese	NA	NA	NA	NA	NA
HL8588	AD	c.1714G > A	p.Asp572Asn			Japanese	NA	NA	NA	NA	NA
HL7492	AD	c.1714G > A	p.Asp572Asn			Japanese	NA	NA	NA	NA	NA
HL3123	Sporadic	c.100C > T	p.Arg34Ter	c.884 + 1G > A	splicing	Japanese	Flat	Profound	No	No	No
HL3604	Sporadic	c.210delG	p.Arg71GlyfsTer5	c.1592A > T	p.Asp531Val	Japanese	Flat	Profound	No	NA	No
HL7927	Sporadic	c.741 + 1_ + 4del	splicing	c.1333C > T	p.Arg445Cys	Japanese	Flat	Severe	NA	NA	No
HL4017	Sporadic	c.1165C > T	p.Arg389Ter	c.1165C > T	p.Arg389Ter	Japanese	Flat	Profound	No	No	Yes
HL8573	AR	c.2047_2048del	p.His683ArgfsTer169	c.2047_2048del	p.His683ArgfsTer169	Japanese	Flat	Profound	NA	NA	No
MED473	Sporadic	c.247_249del	p.Glu83del	c.247_249del	p.Glu83del	Germany	NA	NA	No	No	No
MED214	Sporadic	c.338T > C	p.Met113Thr	c.1534C > T	p.Arg512Ter	Swedish	High freq	Severe	NA	NA	NA
MED131	Sporadic	c.674C > T	p.Pro225Leu	c.1333C > T	p.Arg445Cys	Polish	Flat	Profound	No	No	No
MED097	Sporadic	c.1235delT	p.Met413CysfsTer 4	c.1764G > A	p.Trp588Ter	Polish	High freq	Profound	No	No	No
MED138	AR	c.1764G > A	p.Trp588Ter	c.1764G > A	p.Trp588Ter	Polish	High freq	Profound	No	No	No
MED430	Sporadic	c.2176_2177del	p.Ala726GlufsTer 126	c.2176_2177del	p.Ala726GlufsTer 126	Indian	Flat	Profound	No	No	No

Severity of HL: pure-tone average calculated from the audiometric thresholds at four frequencies (0.5, 1, 2, and 4 kHz) was categorized into mild (PTA: 21–40 dB HL), moderate (41–70 dB HL), severe (71–95 dB HL), or profound (> 95 dB HL)

*AA* amino acid, *AD* autosomal dominant, *AR* autosomal recessive, *NA* not available

*All variants are indicated on NM_138691

The variants identified in this study are summarized in Table 2. In this study, we identified 17 candidate *TMC1* variants, 7 of which were novel variants and 10 were previously reported. Based on ACMG guidelines and ClinGen HLCDWG expert specifications, 5 were classified as “pathogenic” variants and 2 were classified as of “uncertain significance”. Interestingly, *TMC1*:c.1627G > A:p.Asp543Asn variants and *TMC1*:c.1714G > A:p.Asp572Asn variants were identified from 11 and 4 unrelated families with ADNSHL, respectively. Both variants were only identified from ADNSHL patients and were not identified from 6912 autosomal recessive inheritance or sporadic cases, or 2220 unknown family history cases. In addition, these variants were not identified in the gnomAD database or 8.3KJPN (Japanese 8380 genomic variant database). Taken together, the above results strongly supported the pathogenicity of these variants as causative for *TMC1*-associated ADNSHL.

**Table 2:** *TMC1* variants identified in this study

Base change	AA change	Inheritance	SIFT	PP2	MutTaster	REVEL	CADD	8.3KJPN	gnomAD	AD_MAF	AR_MAF	ClinGenHL2018	References
c.100C > T	p.Arg34Ter	AR	–	–	A	–	36	0	0.000056	0	0.00018		Kurima et al. (2002)
c.210delG	p. Arg71GlyfsTer5	AR	–	–	–	–	–	0.0001	0	0	0.00018	Pathogenic	This study
c.247_249del	p.Glu83del	AR	–	–	–	–	–	0	0	0	0.00036		Sloan-Heggen et al. (2016)
c.338 T > C	p.Met113Thr	AR	D	P	D	0.263	24.8	0	0.000004	0	0.00018	VUS	This study
c.674C > T	p.Pro225Leu	AR	T	D	D	0.4	27.2	0	0.000044	0	0.00018		Brownstein et al. (2020)
c.741 + 1_ + 4del	spl	AR	–	–	–	–	–	0	0	0	0.00036	Pathogenic	This study
c.884 + 1G > A	spl	AR	–	–	D	–	27.2	0	0.000012	0	0.00012		Kurima et al. (2002)
c.1165C > T	p.Arg389Ter	AR	–	–	A	–	38	0	0.000068	0	0.00054		Meyer et al. (2005)
c.1235delT	p.Met413CysfsTer4	AR	–	–	-	–	-	0	0	0	0.00018	Pathogenic	This study
c.1333C > T	p.Arg445Cys	AR	D	D	D	0.662	35	0	0.000072	0	0.00036		Sirmaci et al. (2009)
c.1534C > T	p.Arg512Ter	AR	–	–	A	–	42	0	0.0003	0	0.00018		Kurima et al. (2002)
c.1592A > T	p.Asp531Val	AR	D	D	D	0.861	25.7	0	0	0	0.00018	VUS	This study
c.1627G > A	p.Asp543Asn	AD	D	D	D	0.472	32	0	0	0.0082	0		Moteki et al. (2016)
c.1714G > A	p.Asp572Asn	AD	T	D	D	0.465	29.7	0	0	0.0045	0		Kurima et al. (2002)
c.1764G > A	p.Trp588Ter	AR	–	–	A	–	42	0	0.000012	0	0.00054		Tlili et al. (2008)
c.2047_2048del	p.His683ArgfsTer169	AR	–	–	–	–	–	0	0	0	0.00036	Pathogenic	This study
c.2176_2177del	p.Ala726GlufsTer126	AR	–	–	–	–	–	0	0	0	0.00036	Pathogenic	This study

*AA* amino acid, *AD* autosomal dominant, *AR* autosomal recessive, *PP2* PolyPhen2, *MutTaster* Mutation Taster, *AD_MAF* minor allele frequency in ADNSHL cases, *AR_MAF* minor allele frequency in ARNSHL cases

In terms of clinical features, *TMC1*-associated ARNSHL patients showed congenital onset severe-to-profound hearing loss, whereas the *TMC1*-associated ADNSHL patients showed late-onset progressive hearing loss (Table 1). The severity of hearing loss in ADNSHL patients varied from moderate to severe hearing loss depending on patient age. In addition, 3 family members of family #O4886 who carried *TMC1*:c.1627G > A:p.Asp543Asn variants showed normal hearing (Supplemental Fig. 1). Most of the ADNSHL cases complained of the progression of hearing loss and tinnitus; however, only two patients suffered episodes of vertigo.

### Progression of hearing loss in subjects with TMC1-associated ADNSHL

**Fig. 1:**
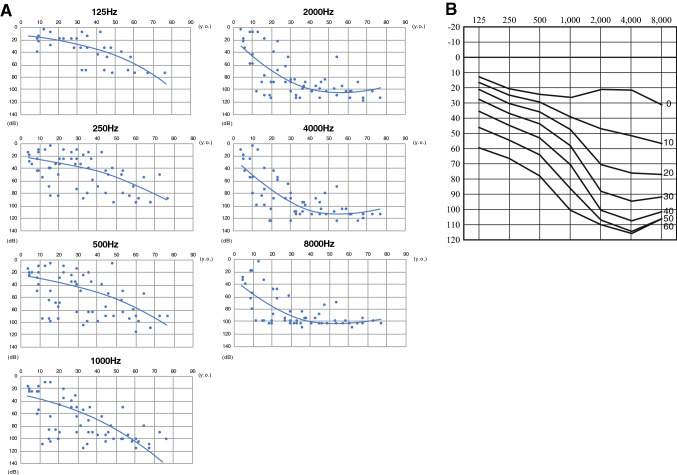
Detailed progression analysis of DFNA36 patients. **A** Hearing thresholds from audiograms (the better ear) of the patients identified in this study and those previously reported were plotted for each frequency. **B** Estimated age-related typical audiogram (ARTA) demonstrating the progression of hearing loss for DFNA36

Most of the *TMC1*-associated ARNSHL patients showed congenital severe-to-profound hearing loss. On the other hand, *TMC1*-associated ADNSHL patients showed late-onset progressive hearing loss (Table 1). To elucidate the progression of hearing deterioration for *TMC1*-associated ADNSHL, we performed regression analysis of age and hearing thresholds of 125, 250, 500, 1000, 2000, 4000 and 8000 Hz (Fig. 1). For this analysis, we used the hearing thresholds for all *TMC1*-associated ADNSHL patients and their affected family members (10 probands and 13 family members) identified in this study and shown in Supplemental Fig. 1. In addition, we also included all available hearing threshold data (34 hearing threshold data) for 24 affected individuals with *TMC1*-associated ADNSHL from previous reports (Kurima et al. 2002; Yang et al. 2010; Zhao et al. 2014; Wang et al. 2018). As shown in Fig. 1, the hearing levels in the higher frequencies deteriorate more rapidly than those in the lower frequencies. The estimated hearing deterioration in terms of pure-tone average (average of 500 Hz, 1000 Hz, 2000 Hz and 4000 Hz) was 1.0 dB per year. The estimated age-related typical audiogram (ARTA) was calculated based on the previously reported method (Huygen et al. 2003) with some modification to allow the use of exponential approximation or logarithmic approximation.

### Haplotype analysis

**Fig. 2:**
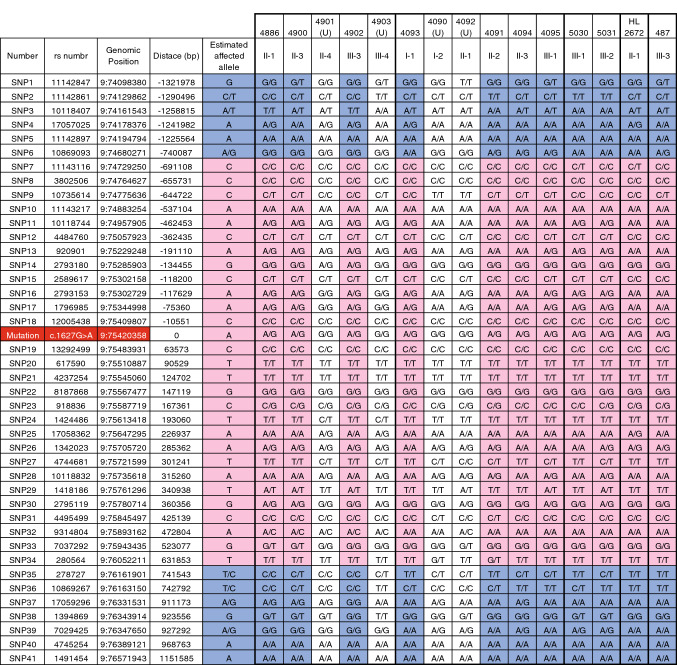
Haplotype analysis of the *TMC1* recurrent variant c.1627G > A:p.Asp543Asn. The estimated haplotypes surrounding the 3 Mbp region of this variant are indicated. The pink area was conserved between unrelated families. The pale blue area was not conserved

Interestingly, 11 unrelated Japanese ADNSHL families carried the same variant (*TMC1*: NM_138691:c.1627G > A:p.Asp543Asn). We, therefore, carried out haplotype analysis to confirm whether this mutation occurred by founder mutation or in a mutational hotspot. Figure 2 shows the haplotype patterns for four unrelated families who carried the same *TMC1*: NM_138691:c.1627G > A variant. As a result, the four unrelated families were found to carry the same haplotype in the 1.3 Mbp region surrounding this mutation (the preserved region ranged from 0.7 Mbp upstream to 0.6 Mbp downstream), suggesting that this mutation occurred and spread as a founder mutation in Japanese populations.

## Discussion

In this study, we identified 26 probands with *TMC1*-associated hearing loss and the prevalence of *TMC1*-associated hearing loss in Japanese hearing loss patients was 0.17% for all patients. The prevalence of *TMC1*-associated hearing loss in other countries is 0.5–8.1% and varies among ethnic populations as described above in the introduction. These differences may be caused by the carrier frequencies of commonly observed mutations. In most previous studies, *TMC1*-associated hearing loss was observed more commonly in ARNSHL patients than in ADNSHL patients, and common mutations which may be caused by founder mutation were involved in these cases. On the other hand, in our Japanese hearing loss cohort, ADNSHL cases were more commonly observed than ARNSHL cases. In addition, all identified variants from Japanese *TMC1*-associated ARNSHL cases differed among patients and no common mutations were identified.

Similar to previous studies, *TMC1*-associated ARNSHL patients showed congenital onset severe-to-profound hearing loss, whereas the *TMC1*-associated ADNSHL patients showed late-onset progressive hearing loss. Indeed, 3 younger agers in family # O4886 showed normal hearing although they carried the same mutation as the other affected family members (Supplemental Fig. 1), supporting the late-onset nature of their hearing loss. In addition, we also clarified the progression of hearing loss for DFNA36 using the hearing threshold data obtained in this study and previous reports, and revealed the hearing deterioration in terms of pure-tone average was 1.0 dB per year. Most of the *TMC1*-associated HL patients identified in this study did not have vestibular symptoms and only two patients had episodes of vertigo. Thus, vestibular symptoms may not be associated with *TMC1*-associated HL cases.

Toward the clinical application of gene therapy for hereditary hearing loss, *TMC1*-associated ADNSHL is believed to be a good candidate, as the late-onset and progressive hearing loss phenotype can be stopped or slowed down by gene therapy prior to hearing deterioration. In addition, ENU-induced model mice with the orthologous mutation identified in human ADNSHL patients are widely used for translational research for gene therapy (Askew et al. 2015; Shibata et al. 2016; Yoshimura et al. 2019; Gao et al. 2018; Nist-Lund et al. 2019; György et al. 2019; Wu et al. 2021). In most of these gene therapy studies, the gene delivering vector, adeno associated virus (AAV), was administrated into the inner ear of neonate mice, allowing prevention of hearing deterioration. However, this timing is equivalent to the developmental stage of the inner ear of the human fetus and makes clinical application difficult. Recently, Yoshimura et al. (2019) reported gene therapy for 2- to 8-week-old mice and prevented hearing deterioration in these model mice, suggesting the appropriate time-window for gene therapy will be wider than previously thought. In this study, we indicated that the hearing deterioration in DFNA36 patients started from their 1st or 2nd decade (teenagers) and this result also supports the notion that the therapeutic time-window for gene therapy to prevent hearing deterioration in human patients might be wider than previously thought.

In this study, we identified 11 unrelated Japanese ADNSHL families that carried same the variant (*TMC1*: NM_138691:c.1627G > A:p.Asp543Asn). Haplotype analysis of *TMC1*: NM_138691:c.1627G > A:p.Asp543Asn showed the same haplotype among the families with the same mutation. This result suggested that this mutation occurred in one common ancestor and was subsequently spread by founder mutation rather than in a mutational hot spot (a mutation which frequently occurs in a specific DNA position). This hypothesis was supported by the fact that this mutation was only identified from Japanese hearing loss patients. This is the first report of a founder mutation identified in DFNA36. Based on the higher prevalence (11 patients carried this mutation in our 11,594 hearing loss subjects), this mutation will be a good candidate for the clinical study of gene therapy for DFNA36. On the other hand, the c.1714G > A:p.Asp572Asn variant observed in this study may be caused by a mutational hotspot. The p.Asp572Asn variant was identified from four Japanese ADNSHL patients in this study, but this variant was also identified from North American, Chinese and Saudi patients (Kurima et al. 2002; Wang et al. 2018; Ramzan et al. 2020; Yuan et al. 2020). The observations of patients from different ethnic backgrounds also support the fact that this variant was caused by a mutational hotspot.

In summary, next-generation sequencing analysis successfully identified 10 previously reported mutations and 7 novel variants for *TMC1*-associated hearing loss. The estimated prevalence of *TMC1*-associated hearing loss in the Japanese hearing loss cohort was 0.17% for all patients, 0.61% for ADNSHL and 0.07% for ARNSHL or sporadic hearing loss cases. This large cohort study of hearing loss patients provided valuable new insights, particularly with regard to hearing deterioration in DFNA36 patients. This information will be useful baseline data for future therapeutics including gene therapy.

## Supplementary Information

Below is the link to the electronic supplementary material.

Supplementary file1 (PDF 522 KB)
