# Supplementary material for: Prevalence and clinical features of autosomal dominant and recessive TMC1-associated hearing loss
Source: Hum Genet. 2021 Sep 14;141(3-4):929–37. doi: 10.1007/s00439-021-02364-2 (PMC9034981; doi:10.1007/s00439-021-02364-2)
Supplement: Supplementary file 1 — Supplementary file1 (PDF 522 KB) [file 439_2021_2364_MOESM1_ESM.pdf]

Prevalence and clinical features of autosomal dominant and recessive *TMC1*-associated hearing loss.

Shin-ya Nishio[1], Shin-ichi Usami[1].

1. Department of Hearing Implant Sciences, Shinshu University School of Medicine, 3-1-1 Asahi Matsumoto, Japan.

Supplemental Figure 1. Pedigree and audiograms for each family with *TMC1* variants. Arrows indicate the probands in each family. Genetic findings for each individual are shown in the pedigree. The age at audiometric testing is shown below the audiogram.

Supplemental Figure 2. Detailed progression analysis of each DFNA36 mutation. Hearing thresholds from audiograms (the better ear) of the patients of each DFNA36 mutation were plotted for each frequency. Estimated age-related typical audiogram (ARTA) demonstrating the progression of hearing loss for each DFNA36 mutation. For p.Gly417Arg mutation in previous reports did not indicate the hearing threshold for 125 Hz, so we excluded from analysis.

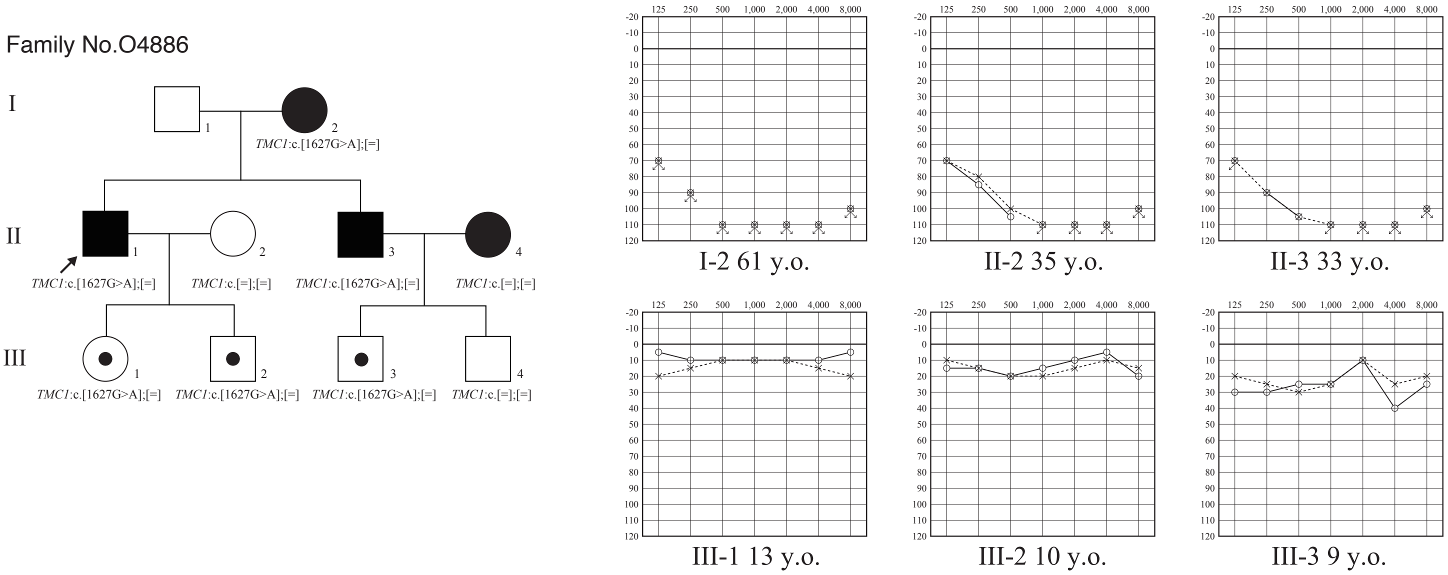
Family No.O4886
I
II
III
TMC1:c.[1627G>A];[=]
TMC1:c.[1627G>A];[=]
TMC1:c.[=];[=]
TMC1:c.[1627G>A];[=]
TMC1:c.[=];[=]
TMC1:c.[1627G>A];[=]
TMC1:c.[1627G>A];[=]
TMC1:c.[1627G>A];[=]
TMC1:c.[=];[=]
I-2 61 y.o.
II-2 35 y.o.
II-3 33 y.o.
III-1 13 y.o.
III-2 10 y.o.
III-3 9 y.o.

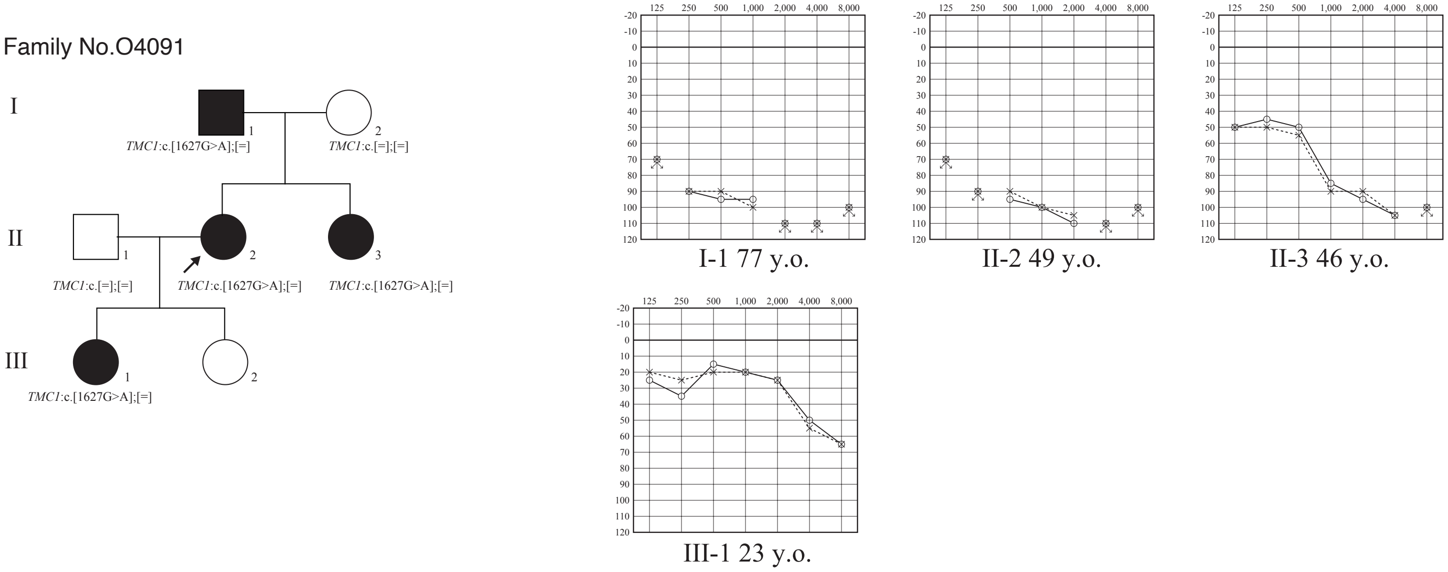
Family No.O4091
I
II
III
TMC1:c.[1627G>A];[=]
TMC1:c.[=];[=]
TMC1:c.[=];[=]
TMC1:c.[1627G>A];[=]
TMC1:c.[1627G>A];[=]
TMC1:c.[1627G>A];[=]
I-1 77 y.o.
II-2 49 y.o.
II-3 46 y.o.
III-1 23 y.o.

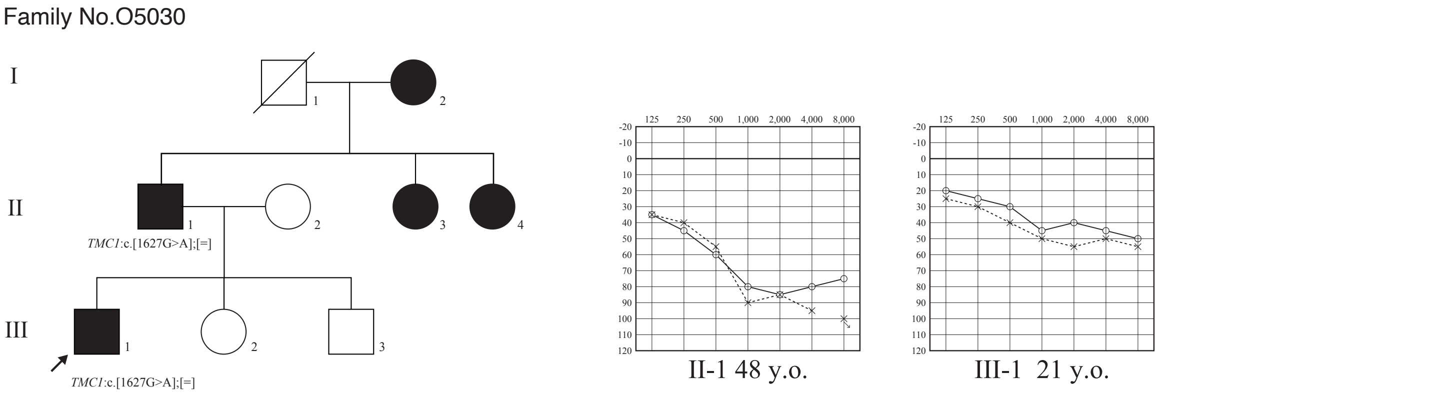
Family No.O5030
I
II
III
TMC1:c.[1627G>A];[=]
TMC1:c.[1627G>A];[=]
II-1 48 y.o.
III-1 21 y.o.

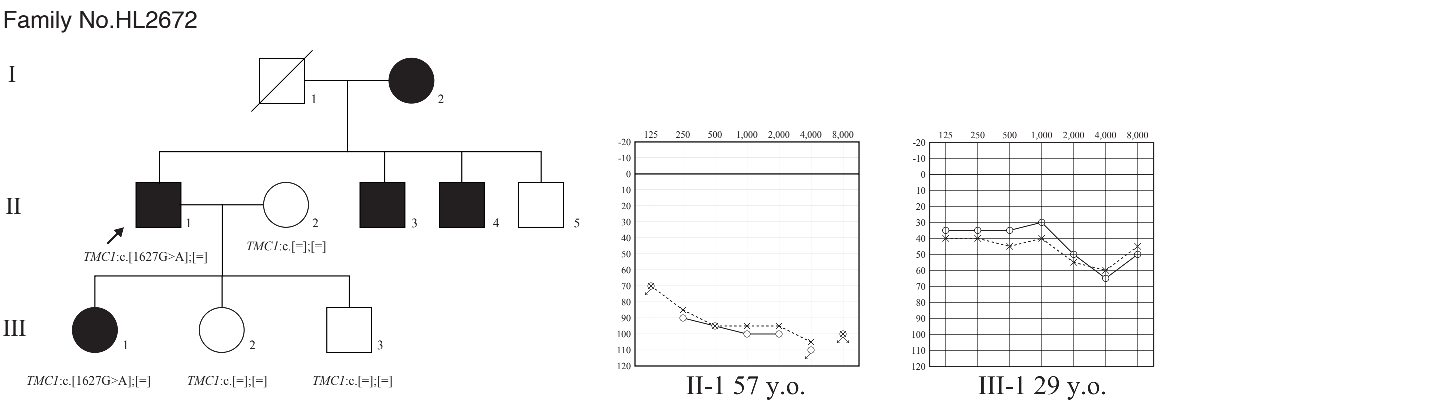
Family No.HL2672
I
II
III
TMC1:c.[1627G>A];[=]
TMC1:c.[=];[=]
TMC1:c.[1627G>A];[=]
TMC1:c.[=];[=]
TMC1:c.[=];[=]
II-1 57 y.o.
III-1 29 y.o.

Family No.O0487

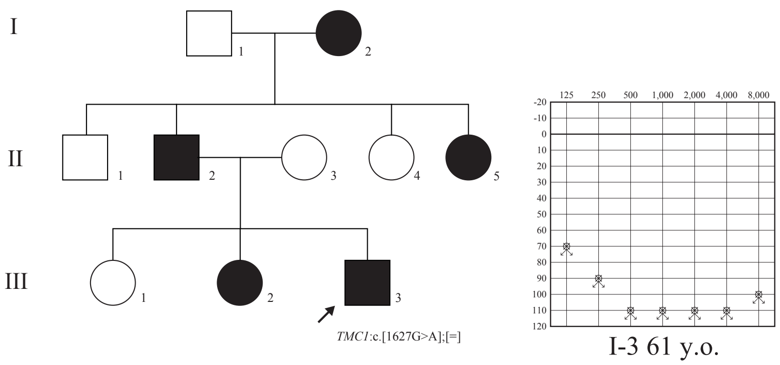

Family No.HL9117

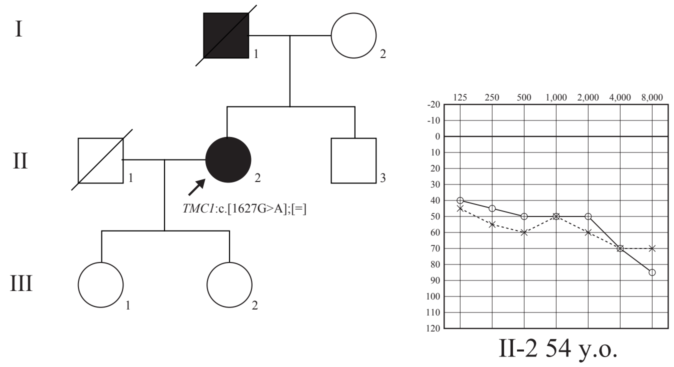

Family No.HL6536

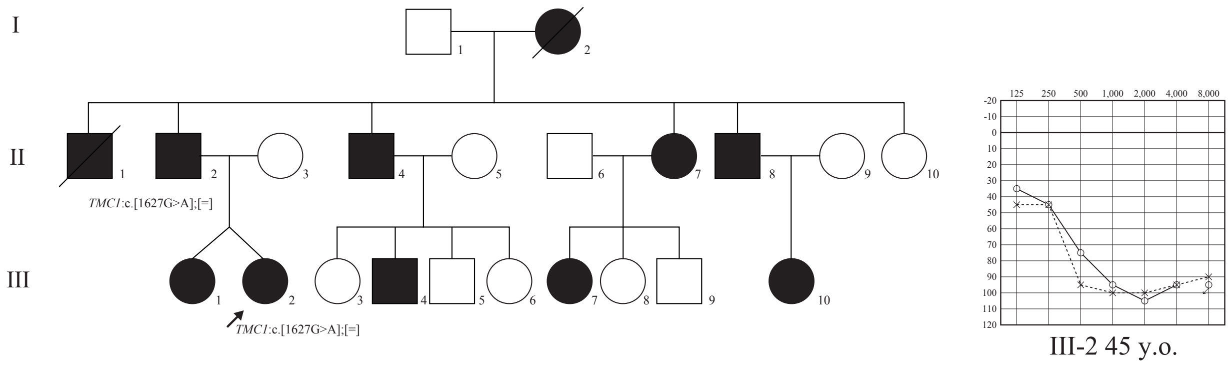

Family No.HL9205

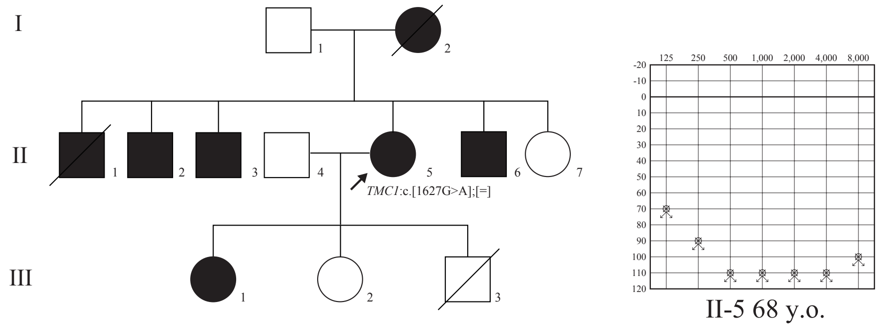

Family No.HL9597

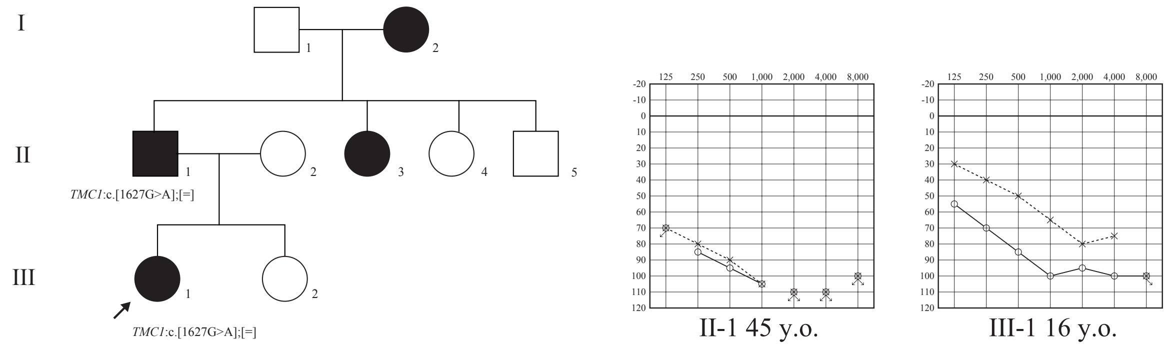

Detailed clinical information for HL4994, HL6717, HL4498, HL8588 and HL7492 were not available.

Family No.HL3819

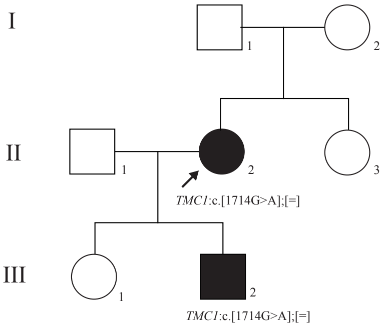

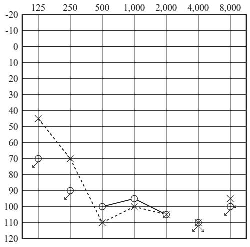

II-2 41 y.o.

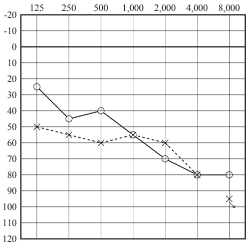

II-2 10 y.o.

Family No.HL3123

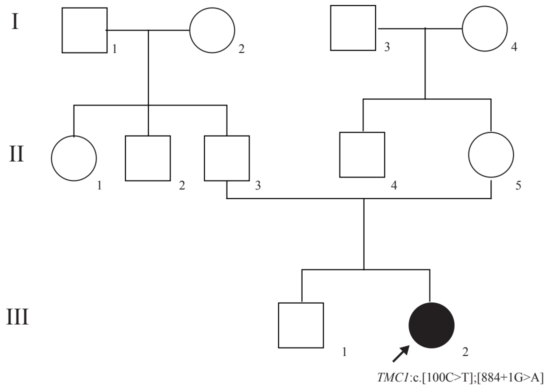

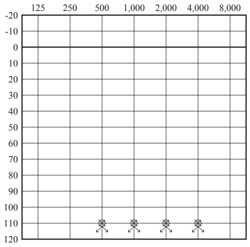

III-2 4 y.o.

Family No.HL3604

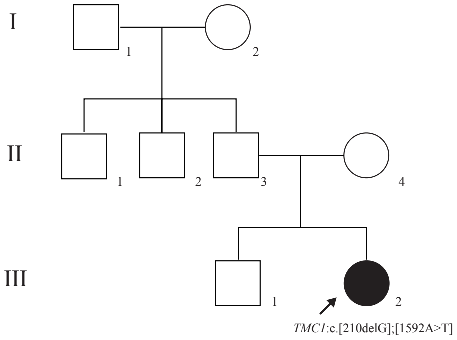

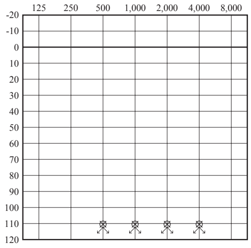

II-3 1 y.o.

Family No.HL7927

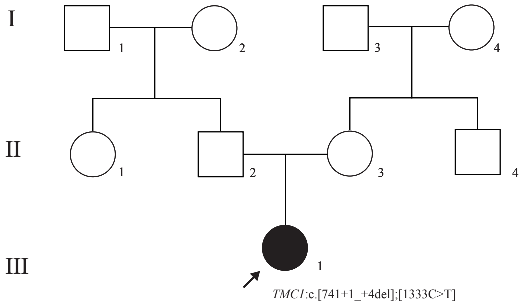

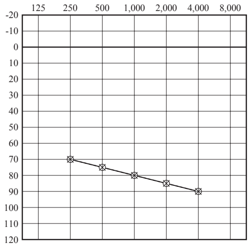

III-1 1 y.o.

Family No.HL4017

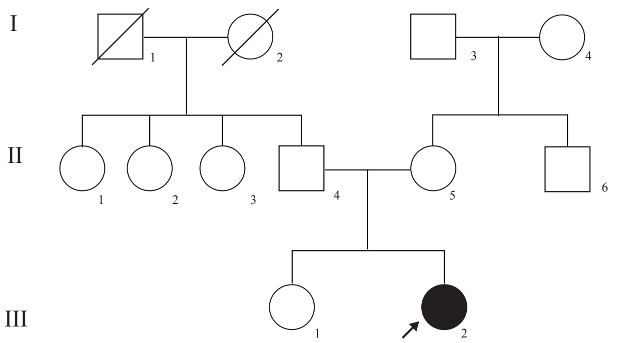

TMC1:c.[1165C>T];[1165C>T]

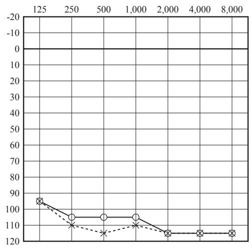

III-2 30 y.o.

Family No.HL8573

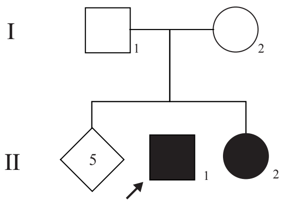

TMC1:c.[2047_2048del];[2047_2048del]

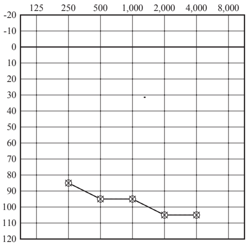

II-2 2 y.o.

Family No.MED097

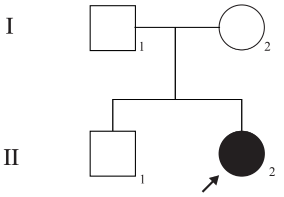

TMC1:c.[1235delT];[c.1764G>A]

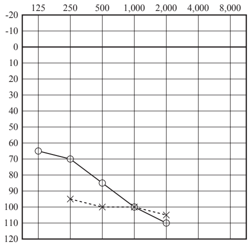

II-2 28 y.o.

Family No.MED473

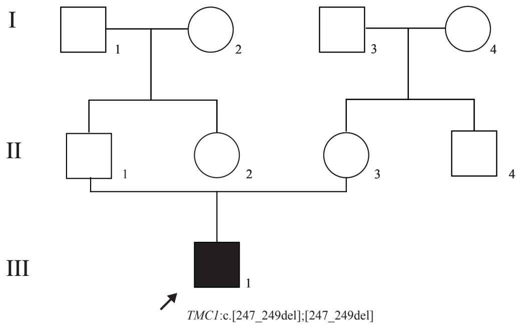

TMC1:c.[247_249del];[247_249del]

NA

Family No.MED214

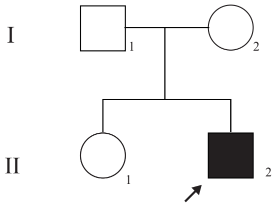

TMC1:c.[338T>C];[1534C>T]

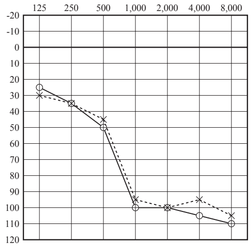

II-2 14 y.o.

Family No.MED138

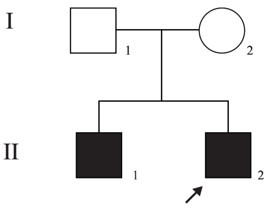

TMC1:c.[1764G>A];[1764G>A]

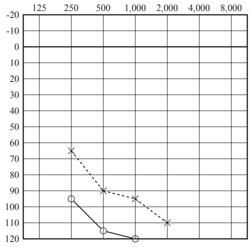

II-2 10 y.o.

Family No.MED131

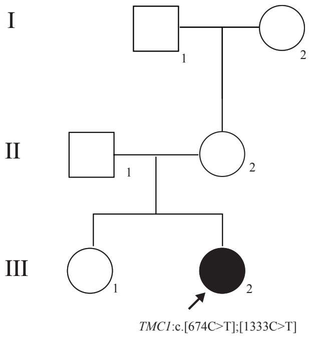

TMC1:c.[674C>T];[1333C>T]

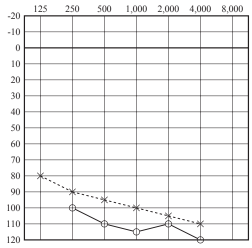

III-2 10 y.o.

Family No.MED430

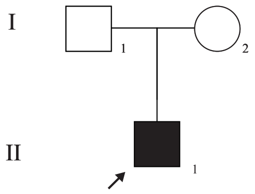

TMC1:c.[2176_2177del];[2176_2177del]

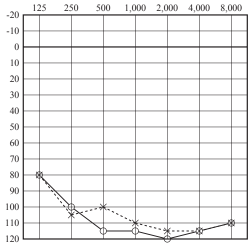

II-1 6 y.o.

p.Asp543Asn

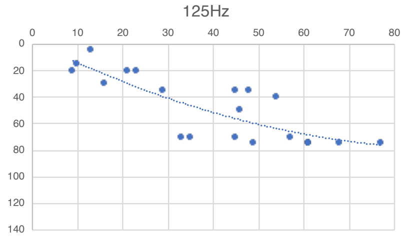
125Hz

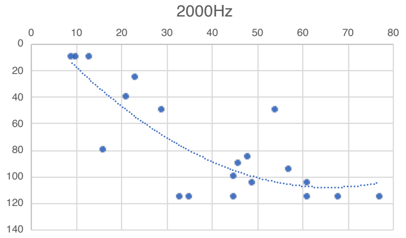
2000Hz

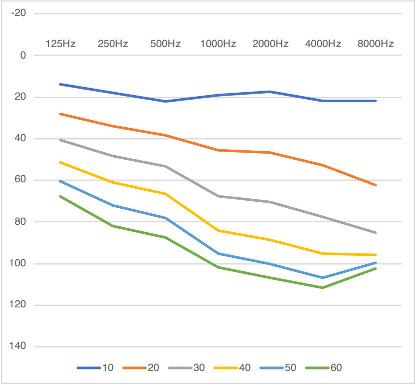
-20
125Hz
250Hz
500Hz
1000Hz
2000Hz
4000Hz
8000Hz
10
20
30
40
50
60

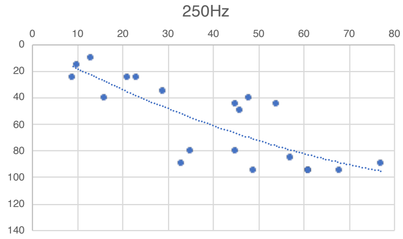
250Hz

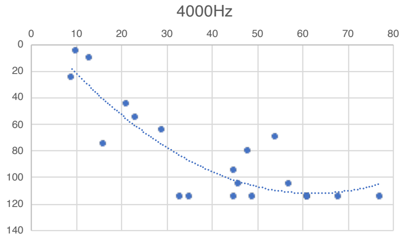
4000Hz

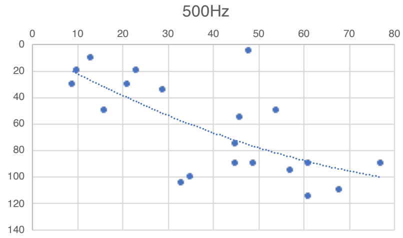
500Hz

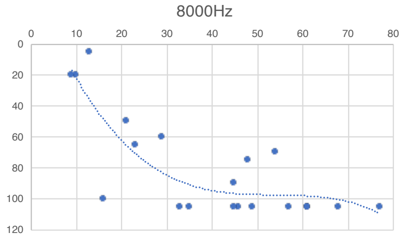
8000Hz

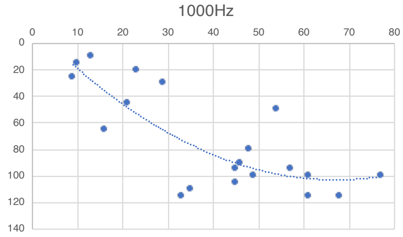
1000Hz

p.Met418Lys

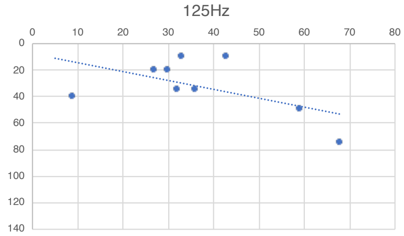
125Hz

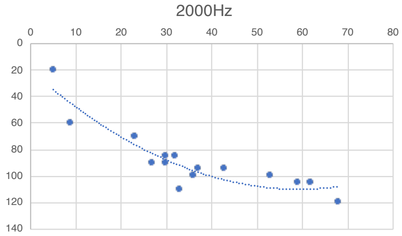
2000Hz

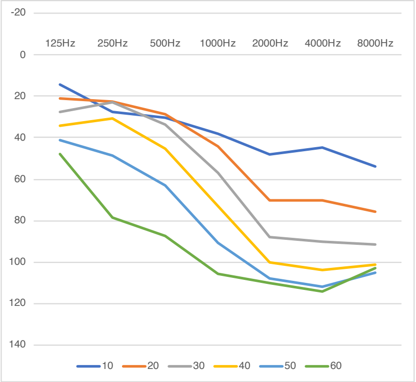
125Hz
250Hz
500Hz
1000Hz
2000Hz
4000Hz
8000Hz
10
20
30
40
50
60

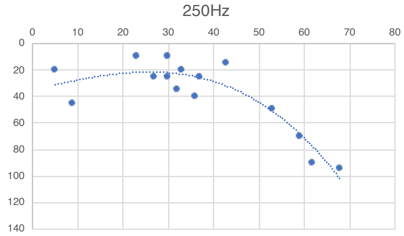
250Hz

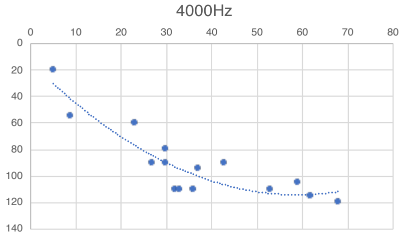
4000Hz

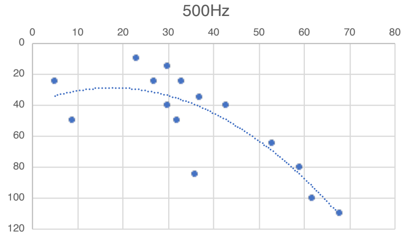
500Hz

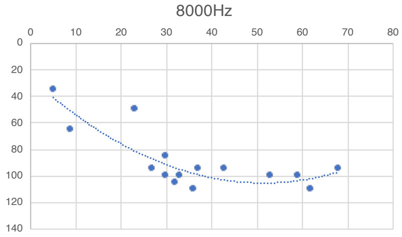
8000Hz

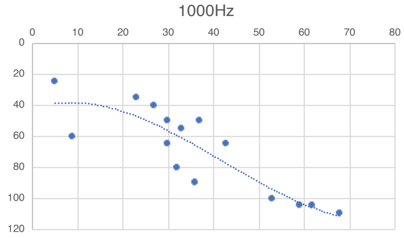
1000Hz

p.Asp572Asn

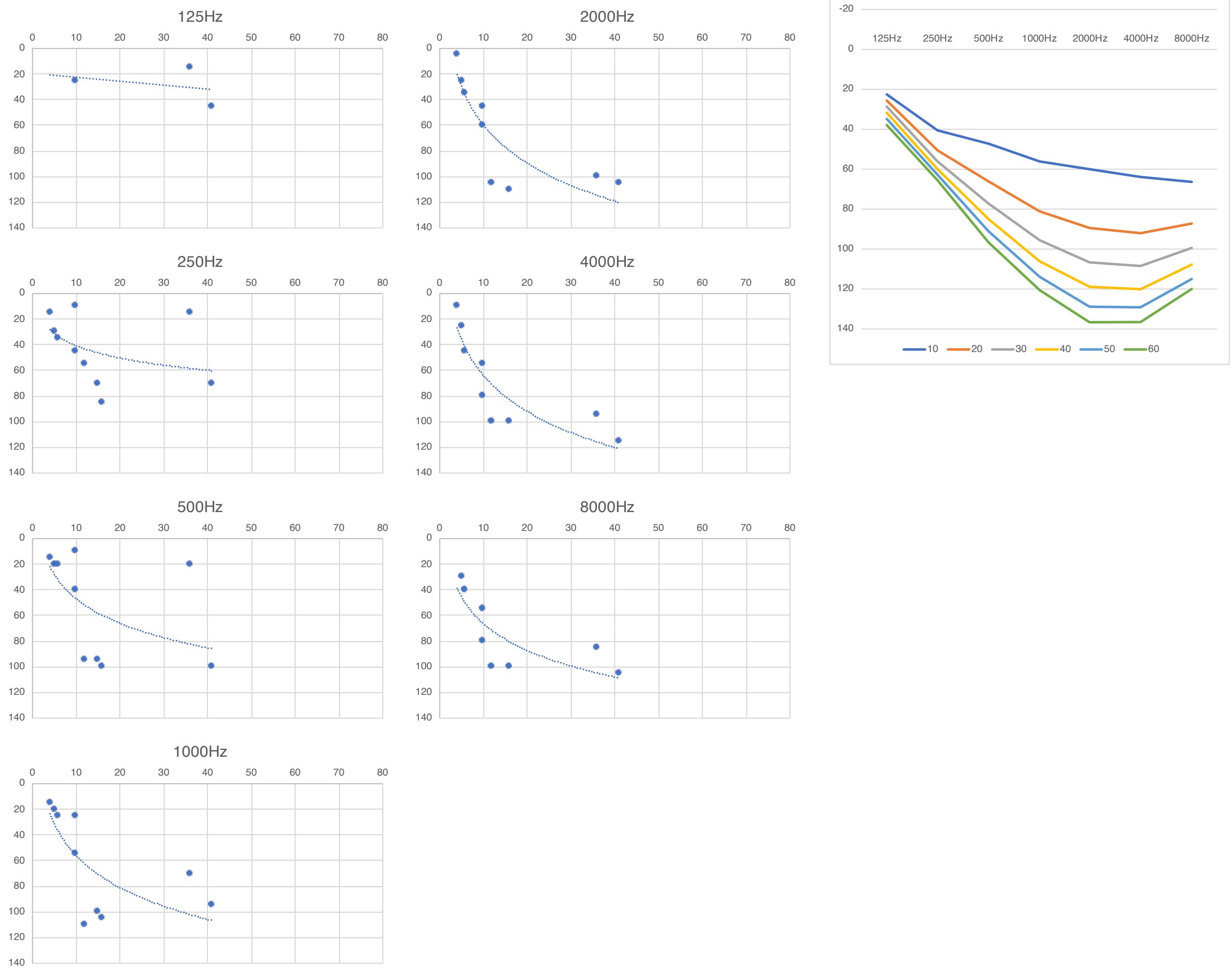
125Hz
2000Hz
250Hz
4000Hz
500Hz
8000Hz
1000Hz
0 10 20 30 40 50 60 70 80
0 20 40 60 80 100 120 140
125Hz 250Hz 500Hz 1000Hz 2000Hz 4000Hz 8000Hz
-20 0 20 40 60 80 100 120 140
10 20 30 40 50 60
